# Associations of handgrip strength with all-cause and cancer mortality in older adults: a prospective cohort study in 28 countries

**DOI:** 10.1093/ageing/afac117

**Published:** 2022-05-25

**Authors:** Rubén López-Bueno, Lars Louis Andersen, Joaquín Calatayud, José Casaña, Igor Grabovac, Moritz Oberndorfer, Borja del Pozo Cruz

**Affiliations:** Department of Physical Medicine and Nursing, University of Zaragoza, Zaragoza, Spain; National Research Centre for the Working Environment, Copenhagen, Denmark; Exercise Intervention for Health Research Group (EXINH-RG), Department of Physiotherapy, University of Valencia, Valencia, Spain; National Research Centre for the Working Environment, Copenhagen, Denmark; National Research Centre for the Working Environment, Copenhagen, Denmark; Exercise Intervention for Health Research Group (EXINH-RG), Department of Physiotherapy, University of Valencia, Valencia, Spain; Exercise Intervention for Health Research Group (EXINH-RG), Department of Physiotherapy, University of Valencia, Valencia, Spain; Department of Social and Preventive Medicine, Centre for Public Health, Medical University of Vienna, Vienna, Austria; Department of Social and Preventive Medicine, Centre for Public Health, Medical University of Vienna, Vienna, Austria; Centre for Active and Healthy Ageing, Department of Sports Science and Clinical Biomechanics, University of Southern Denmark, Odense, Denmark

**Keywords:** ageing, longevity, longitudinal, physical exercise, physical activity, older people

## Abstract

**Background:**

mixed evidence exists on the association between muscle strength and mortality in older adults, in particular for cancer mortality.

**Aim:**

to examine the dose–response association of objectively handgrip strength with all-cause and cancer mortality.

**Study Design and Setting:**

data from consecutive waves from the Survey of Health, Ageing and Retirement in Europe comprising 27 European countries and Israel were retrieved. Overall, 54,807 men (45.2%; 128,753 observations) and 66,576 women (54.8%; 159,591 observations) aged 64.0 (SD 9.6) and 63.9 (SD 10.2) years, respectively, were included. Cox regression and Fine-Grey sub-distribution method were conducted.

**Results:**

during the follow-up period (896,836 person-year), the fully adjusted model showed the lowest significant risk estimates for the highest third of handgrip strength when compared with the first third (reference) in men (hazard ratio [HR], 0.41; 95% confidence interval [CI], 0.34–0.50) and women (HR, 0.38; 95% CI, 0.30–0.49) for all-cause mortality. We identified a maximal threshold for reducing the risk of all-cause mortality for men (42 kg) and women (25 kg), as well as a linear dose–response association in participants aged 65 or over. No robust association for cancer mortality was observed.

**Conclusion:**

these results indicate an inverse dose–response association between incremental levels of handgrip and all-cause mortality in older adults up to 42 kg for men and 25 kg for women, and a full linear association for participants aged 65 years or over. These findings warrant preventive strategies for older adults with low levels of handgrip strength.

## Key Points

- Up to a threshold of 42 kg in men and 25 kg in women, increases in handgrip strength reduce the risk of all-cause mortality.
- Older participants showed an inverse linear dose–response relationship between handgrip strength and all-cause mortality.
- No consistent association was observed for cancer-specific mortality.

## Introduction

Handgrip strength is simple to assess, does not require specific environmental conditions, and the equipment involved is inexpensive and portable. Handgrip strength is also considered a reliable biomarker in older adults [1]; previous studies have reported declines in handgrip strength with ageing at an annual rate of $\sim$1% after midlife, and higher handgrip strength at midlife has been suggested to increase resilience to ageing [2, 3]. A substantial loss of handgrip strength may indicate both health deterioration and early ageing leading to an increased risk of future disability and morbidity, particularly among older adults [1, 4, 5].

There is evidence supporting an inverse association between handgrip strength and all-cause mortality. A meta-analysis with 2,000,000 participants found higher levels of handgrip strength to be associated with lower risk of all-cause mortality, irrespective of age and length of follow-up [6]. Studies also show consistent associations of handgrip strength with cardiovascular mortality [4, 7, 8]. Nonetheless, the association of handgrip strength with cancer mortality remains controversial [4, 7, 9–11]. A recent study involving older Chinese adults showed that higher handgrip strength was associated with lower risk of cancer mortality, although this observation was only confirmed for lung and colorectal cancer in men and breast cancer in women [9]. Other studies found no association between handgrip strength and cancer mortality [4, 11]. However, these studies were based on single timepoint measurements of handgrip strength, which could lead to serious bias of their estimates, and are likely subjected to reverse causality. Therefore, studies with repeated measures are sorely needed to prove the predictive ability of handgrip strength for stratifying mortality risks due to all causes and due to cancer.

This study aims to examine the association of handgrip strength with all-cause and cancer mortality in a large representative sample of older adults from 28 countries with repeated measurements of handgrip. A secondary aim was to determine the shape of the dose–response association between grip strength and mortality.

## Methods

### Study design and population

**Figure 1:**
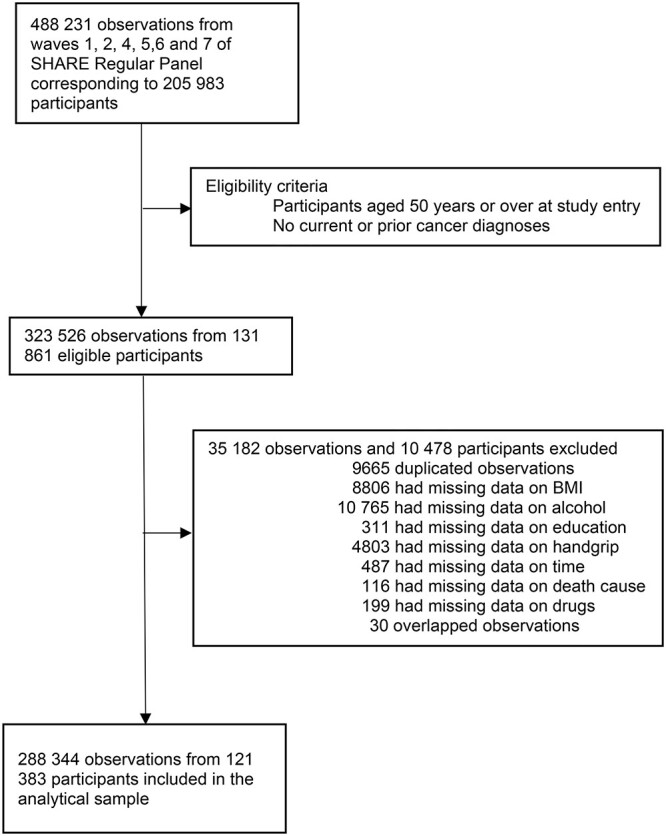
Study profile.

This study included data from waves 1, 2, 4, 5, 6 and 7 from the Survey of Health, Ageing and Retirement in Europe (SHARE), a biannual survey currently recruiting individuals aged 50 or older from European countries and Israel [12, 13]. We did not consider Wave 3 in the current study because information on the exposure of interest (i.e. handgrip strength) was not provided [14]. More details are given in Supplementary Data, Appendix 1. Figure 1 shows more descriptive details of the study sample. This study received the approval of the Ethics Committee of Research in Humans of the University of Valencia (registered code 1510464) and was reported according to Strengthening the Reporting of Observational Studies in Epidemiology [15].

Handgrip strength was measured twice for each hand using a handheld dynamometer (Smedley, S Dynamometer, TTM, Tokyo, 100 kg). Participants were followed throughout the study period to determine whether they were dead or alive. In case of death, information regarding both date and cause of death was obtained from a proxy interview with a relative, a household member, a neighbour or any other person close to the deceased participant using a standardised end-of-life interview.

Based on a literature review on the topic [16–20], we explored potential causal and confounding pathways between handgrip and all-cause and specific-cancer mortality using a directed acyclic graph (Supplementary Data Figure S1). Further details on the study variables are provided in Supplementary Data, Appendix 2.

### Statistical analyses

We conducted all statistical analyses in Stata version 16.1 (StataCorp, Texas, USA). We used a Cox regression with both time-varying exposure and covariates to estimate the hazard ratios (HRs) for all-cause mortality. In addition, the Fine-Grey subdistribution method accounting for competing risk (i.e. mortality due to other causes than cancer) was used to estimate subhazard ratios (SHRs) for cancer-specific mortality. Time-on-study in months was used as the timescale. Handgrip strength was categorised into sex-specific thirds based on handgrip strength values at study entry and the lowest third served as reference.

We examined the proportional hazards assumption by testing interactions with log(time) using stphplot command and found no evidence of assumption violation. After assessing interactions between handgrip and several covariates, we detected a significant interaction concerning sex (chunk test), thus we conducted all our analyses stratified by sex. Two models were tested; a model with age at the time of the interview as confounder (Model A) and a fully adjusted model (Model B) that included age, country, education, body mass index, alcohol and drug consumption as confounders. All the analyses accounted for the survey design and were weighted according to each country population. The results were visualised as forest plots. In addition, we assessed the dose–response associations of handgrip strength (modelled as a continuous exposure) with all-cause and cancer-specific mortality using restricted cubic splines to allow for potential nonlinearity. For this analysis, we trimmed observations <5% and greater than 95% of the distribution and prespecified knots placed at the 10th, 50th (reference) and 90th percentiles of the exposure distribution [21]. We assumed linearity for values below the 10th percentile and for values above the 90th percentile. Departure from linearity was assessed by a Wald test examining the null hypothesis that the coefficient of the second spline was equal to zero. Results are reported as either HRs or SHRs ratios with 95% confidence intervals (CIs) and levels of significance were set at *P* < 0.05. Details on conducted sensitivity analyses are given in Supplementary Data, Appendix 3.

## Results

### Demographics

The final sample included 54,807 men (45.2%; 128,753 observations) and 66,576 women (54.8%; 159,591 observations) with complete data on exposure and covariates of interest. The mean age was 64.0 (SD 9.6) and 63.9 (SD 10.2) years at study entry for men and women, respectively (Table 1). During a median (interquartile range) of 7.4 years of follow-up (2.7–8.0) and 896,836 person-year, 2,675 (4.5%) men and 2,091 (3.1%) women died due to all-causes, whereas 731 (1.3%) women and 540 (0.8%) men died due to cancer. Mean values of handgrip strength differed between men [42.6 kg (SD 11.1)] and women [27.1 kg (SD 7.8)] (Table 1).

**Table 1:** Characteristics of study participants at study entry by sex

	Men = 54,807 (45.2%)		Women = 66,576 (54.8%)	
*N* = 121,383	*n* (%)	Mean (SD)	*n* (%)	Mean (SD)
**Age** (y)		64.0 (9.6)		63.9 (10.2)
**Body mass index**				
Underweight (<18.5 kg/m^2^)	224 (0.4)		1,071 (1.6)	
Normal (18.5– < 25 kg/m^2^)	16,890 (30.8)		26,341 (39.6)	
Overweight (25– < 30 kg/m^2^)	26,452 (48.3)		24,457 (36.7)	
Obese (≥30 kg/m^2^)	11,241 (20.5)		14,707 (22.1)	
**Education** ^**a**^				
None	2,022 (3.7)		2,995 (4.5)	
Preprimary	9,054 (16.5)		13,199 (19.8)	
Primary	8,830 (16.1)		12,764 (19.2)	
Lower secondary	19,778 (36.1)		21,484 (32.3)	
Upper secondary	2,566 (4.7)		3,051 (4.6)	
Postsecondary non-tertiary	11,818 (21.6)		12,470 (18.6)	
First stage of tertiary	533 (1.0)		325 (0.5)	
Second stage of tertiary	23 (0.0)		48 (0.1)	
Other	183 (0.3)		240 (0.4)	
**Alcohol consumption**				
Almost every day	12,552 (22.9)		6,533 (9.8)	
Five or 6 days a week	1,930 (3.5)		1,076 (1.6)	
Three or 4 days a week	5,361 (9.8)		4,187 (6.3)	
Once or twice a week	10,749 (19.5)		10,413 (15.6)	
Once or twice a month	6,109 (11.2)		8,830 (13.3)	
Less than once a month	3,831 (7.0)		7,551 (11.3)	
Not at all in the last 6 months	14,275 (26.1)		27,986 (42.1)	
**Drug consumption**				
Any	17,977 (32.8)		17,843 (26.8)	
None	36,830 (67.2)		48,733 (73.2)	
**Country**				
Austria	2,527 (4.6)		3,360 (5.1)	
Belgium	4,118 (7.5)		4,713 (7.1)	
Bulgaria	803 (1.5)		1,050 (1.6)	
Croatia	9 (0.0)		7 (0.0)	
Czech Republic	3,448 (6.3)		4,558 (6.8)	
Denmark	2,552 (4.6)		2,850 (4.3)	
Estonia	3,000 (5.5)		4,269 (6.4)	
Finland	828 (1.5)		920 (1.4)	
France	3,357 (6.1)		4,182 (6.2)	
Germany	3,764 (6.7)		4,197 (6.2)	
Greece	2,269 (4.1)		2,846 (4.3)	
Hungary	1,238 (2.3)		1,603 (2.4)	
Ireland	439 (0.8)		500 (0.8)	
Israel	1,577 (2.9)		1,872 (2.8)	
Italy	3,495 (6.4)		4,019 (6.0)	
Latvia	588 (1.1)		983 (1.5)	
Lithuania	667 (1.2)		1,172 (1.8)	
Luxembourg	917 (1.7)		1,039 (1.6)	
Malta	461 (0.8)		561 (0.8)	
Netherlands	2,758 (5.0)		3,144 (4.7)	
Poland	2,548 (4.7)		3,003 (4.5)	
Portugal	881 (1.6)		1,060 (1.6)	
Romania	858 (1.6)		1,118 (1.7)	
Slovakia	929 (1.7)		1,034 (1.6)	
Slovenia	2,220 (4.1)		2,768 (4.2)	
Spain	3,711 (6.8)		4,233 (6.3)	
Switzerland	1,994 (3.6)		2,328 (3.5)	
Sweden	2,881 (5.3)		3,217 (4.8)	
**Handgrip strength (kg)**	42.6 (11.1)		27.1 (7.8)	
**Handgrip strength thirds**				
1	18,422 (33.6)		23,313 (35.0)	
2	19,524 (35.6)		23,147 (34.8)	
3	16,861 (30.8)		20,116 (30.2)	
**All-cause mortality**				
Yes	2,675 (4.5)		2,091 (3.1)	
No	52,132 (95.5)		64,485 (96.9)	
**Cancer mortality**				
Yes	731 (1.3)		540 (0.8)	
No	54,076 (98.7)		66,036 (99.2)	

^a^Based on ISCED 1997 classification.

### All-cause mortality

**Figure 2:**
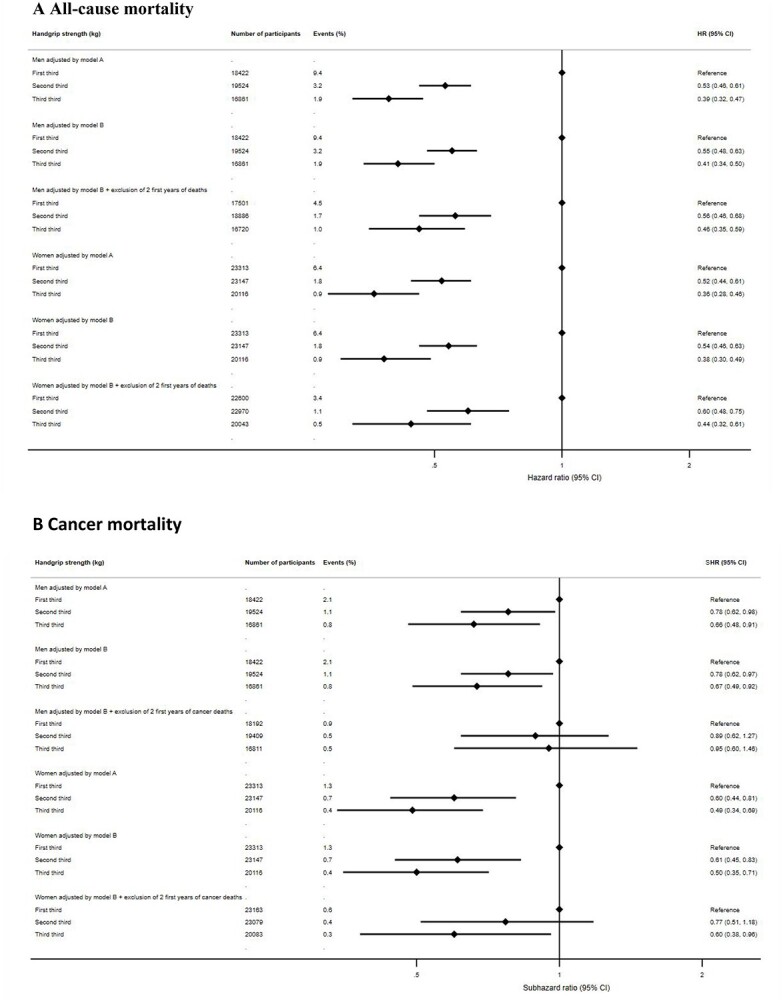
Prospective associations between handgrip strength and mortality by sex. (A) All-cause mortality. (B) Cancer mortality Model A adjusted for age. Model B Adjusted for age, education, country, body mass index, drug and alcohol consumption.

Results from the model adjusted for age only (Model A) showed that higher levels of handgrip strength were associated with a lower risk of all-cause mortality in men and women (Figure 2). In men, the lowest risk was observed for participants in the highest third of handgrip strength (HR, 0.39; 95% CI, 0.32–0.47) compared with participants in the first third (i.e. reference). For women, a significant lower risk of all-cause mortality was also observed for the highest third of handgrip strength (HR, 0.36; 95% CI, 0.28–0.46).

The observed associations were consistent in the fully adjusted model (Model B), and the lowest significant risk estimates were also observed for the highest third of handgrip strength when compared with the first third (reference) in men (HR, 0.41; 95% CI, 0.34–0.50) and women (HR, 0.38; 95% CI, 0.30–0.49) (Figure 2).

**Figure 3:**
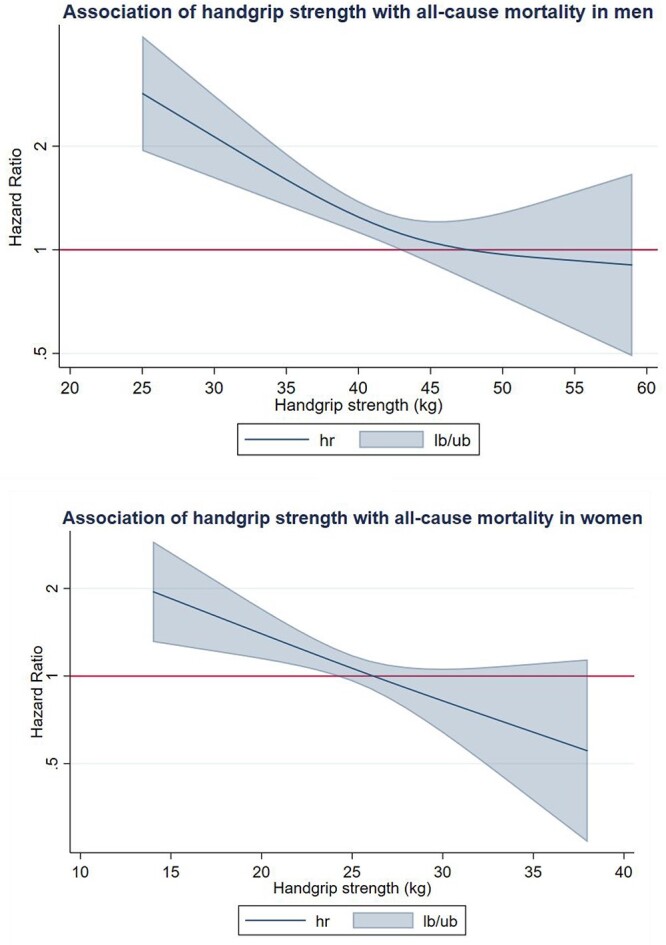
Dose–response association (Adjusted HRs and associated 95% confidence interval band) between handgrip strength (kg) and all-cause mortality in men and women. Adjusted for Model B (age, education, country, body mass index, drug and alcohol consumption) and exclusion of all-cause deaths of two first years of follow-up.

The fully adjusted model using handgrip strength as a continuous exposure (i.e. spline modelling) showed, in a linear dose–response fashion, higher all-cause mortality risk for both men and women with lower handgrip strength values than the median (i.e. for values below 43 kg and 26 kg for men and women, respectively). However, we did not detect significant associations with all-cause mortality in participants with higher handgrip strength values than the median in either men or women (Figure 3).

### Cancer mortality

Men and women in the middle and highest third of handgrip strength had a lower risk of cancer mortality compared with participants in the first third (reference) in Model A (Figure 2). Men categorised in the highest third of handgrip strength exhibited the lowest risk of cancer mortality (SHR, 0.66; 95% CI, 0.48–0.91) when compared with the reference group (i.e. lowest third). Among women, a significantly lower risk of cancer mortality was also observed for participants in the highest third of handgrip strength (SHR, 0.49; 95% CI, 0.34–0.69).

The fully adjusted model (Model B) yielded consistent results, and participants in the highest third of handgrip strength had the lowest risk when compared with the lowest third (reference) in men (SHR 0.67, 95% CI, 0.49–0.92) and women (SHR 0.50; 95% CI, 0.35–0.71) (Figure 2).

**Figure 4:**
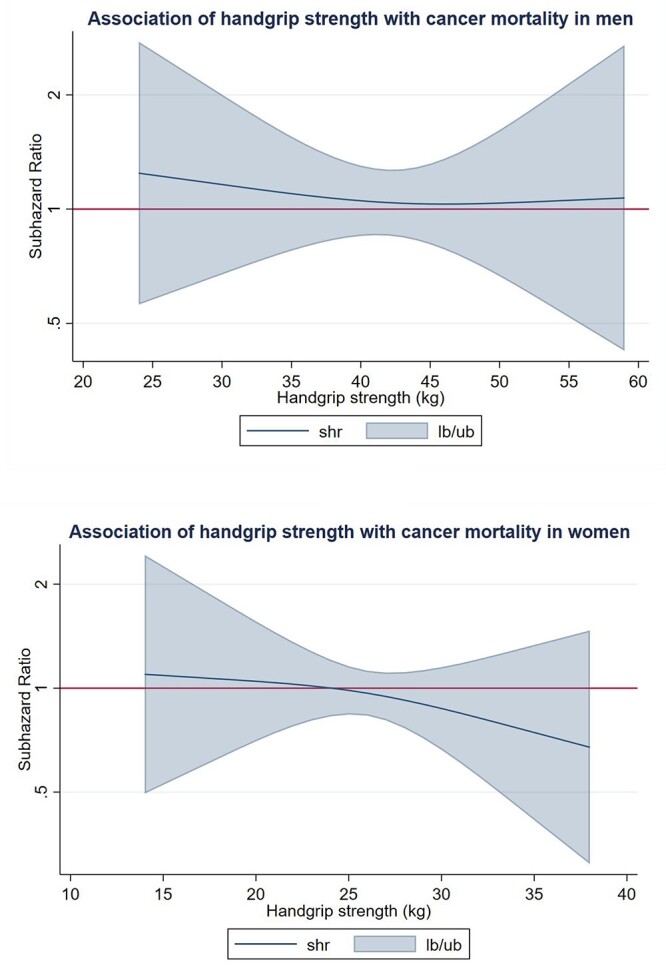
Dose–response association (Adjusted SHRs and associated 95% confidence interval band) between handgrip strength (kg) and cancer mortality in men and women. Adjusted for Model B (age, education, country, body mass index, drug and alcohol consumption) and exclusion of cancer deaths of two first years of follow-up.

We did not observe significant associations between handgrip strength and cancer mortality when using restricted cubic spline models (Figure 4).

Information on results regarding sensitivity analyses are provided in Supplementary Data, Appendix 4.

## Discussion

The results of our study indicate the existence of a linear inverse association between incremental levels of handgrip and risk of all-cause mortality in older adults up to 42 kg for men and 25 kg for women, and a full linear dose–response association was observed for a subgroup of older men and women (≥65 years) within specific handgrip strength ranges. Interestingly, higher levels of handgrip strength were associated with a lower risk for all-cause mortality up to 42 kg and 25 kg in men and women, respectively, but no minimal or maximal threshold was identified for older individuals, who showed an all-cause mortality risk reduction for each kilogram increase of handgrip strength. The same upper thresholds were also observed for overweight and obese individuals together, although could not be fully confirmed for a subgroup of underweight and normal body mass index categories. These results shed light on the little-known dose–response association between handgrip and all-cause mortality in older adults, which has been mainly investigated by categorising handgrip strength instead of using it as a continuous variable [4, 22].

Unexpectedly, handgrip strength did not reduce cancer mortality risk after robust analyses and linear dose–response examination, which was observed in both men and women. Similar to previous studies [4, 10], we could not confirm a consistent association between handgrip strength and mortality due to cancer.

Our results shed light into the ongoing discussions on the utility of handgrip strength as an ageing biomarker and could inform clinical guidelines to detect older men and women at risk of premature mortality. Despite the differences between men and women in relation to handgrip strength values, we observed similar associations for both sexes concerning all-cause and cancer mortality. Furthermore, comparable trajectories were also observed in dose–response analyses for the two examined outcomes among men and women, which indicates the possibility of common pathways leading to reducing all-cause mortality risk irrespective of baseline handgrip strength levels. In contrast, prior research observed stronger associations among older women than those observed in men [22], a difference possibly attributed to hormonal factors related to gaining in muscle strength associated with women solely [23].

### All-cause mortality

Our findings agree with observations from prior research highlighting a higher risk of all-cause mortality among Korean older men and women with low levels of handgrip strength [4, 24]. Consistent results have also been observed in older adults from Chile [25], Mexico [26]^,^ and Africa [27]. Similar to prior research, this study also found some sex differences in the magnitude of the association between handgrip strength and all-cause mortality, with men reducing more the risk with higher levels of handgrip strength than women [10]. It is plausible that handgrip strength may have a different role for mortality in men and women [28, 29].

Unlike previous research [11], we did not find a minimal threshold for the inverse association of handgrip strength with mortality. This is important as it suggests that even low levels of handgrip strength may result in higher longevity. We also observed that our derived thresholds for maximal longevity gains with increasing handgrip strength are similar than those reported as references for older European adults [29]. Interestingly, our spline modelling revealed that low levels of handgrip strength are a consistent risk factor, whereas very high levels of handgrip strength may not provide additional protection to all-cause mortality compared with having a moderate level of strength. Together, our study supports the need for targeting older adults with low levels of handgrip strength as a way to improve longevity of older adults. The derived thresholds in this study could serve as referent for target.

### Cancer mortality

Consistent with previous studies [10, 11], this study did not confirm an association between handgrip strength and cancer mortality among older adults. By contrast, other studies have suggested a significant inverse association betweeen handgrip strength and cancer mortality in both men and women, although the magnitude of the observed association was weak [7, 9]. Detailed examination of mortality due to specific cancer-types has revealed different results. For instance, although significant associations were observed for colorectal, lung and breast cancers, this was not the case for prostate cancer [7, 9]. The aetiology of different cancer types may partly explain the different results across studies, including ours [30]. Interestingly, a retrospective investigation with cancer patients observed a higher mortality risk among participants with handgrip strength values below previously estimated cut-off points; and such observation was particularly relevant for breast cancer in women and for lung and colorectal cancer in men [9]. Similarly, patients with incurable cancer and very low levels of handgrip strength have shown an increased mortality risk [31]. It is possible that higher levels of handgrip strength do not equally reduce risk for all types of cancer, and that differences found in other studies can also reflect different sample features comprising either cancer patients or individuals without a prior cancer diagnosis. Further investigation to disentangle the role and pathways by which handgrip strength may impact different types of cancer mortality is warranted. For example, a previous study reported that higher handgrip strength levels were associated with lower inflammatory markers [32]. Because inflammatory markers such as C-reactive protein have been associated with both cancer diagnoses and mortality [33–35], the association between higher handgrip strength and lower specific cancer mortality via reduction of such inflammatory marker seems plausible and deserves exhaustive investigation.

### Strengths and limitations

Key strengths of this study are the use of a large and representative sample from 28 countries with an objective measure of handgrip strength. Another relevant strength is the use of time-varying exposure, outcomes and covariates in our modelling strategy, which reduces the possibility of bias of our estimates. We also accounted for competing risks in our estimations of cancer-specific mortality outcomes. Our handgrip measurement allowed us to model the continuous dose–response association between handgrip strength and mortality outcomes, which is critical to inform clinical practice (i.e. determination of relevant thresholds and shape of the dose–response association). Such association showed a significant linear dose–response fashion in older adults (≥65 years) for both sexes, which may indicate that gaining strength is particularly relevant to increase longevity at older ages. Concerning body mass index, the identified thresholds were also observed for overweight and obese individuals together but did not remain as significant for the subgroup comprising underweight and normal weight categories. Importantly, we also took measures to minimise the chance of reverse causation (i.e. lower handgrip strength as result of the course of the disease) by removing all the events that occurred during the first 2 years of follow-up. Furthermore, substantial differences regarding median handgrip strength values in the overall trajectories between survivor and nonsurvivor participants strengthen our findings; however, important fluctuations due to less participants with longer follow-up periods make these estimations particularly unstable in the final stages.

Our study should also be considered in light of several limitations. First, due to the large number of participants with missing values concerning smoking habits, fruit and vegetable consumption, and physical inactivity, such variables were not included in the main analyses, which could increase the likelihood of residual confounding. However, additional analyses examining potential residual confounding bias through E-values showed a broad margin for residual confounding for the significant associations observed in such analyses (Supplementary data Tables S2 and S3), which give us confidence on our estimates. Also, the sensitivity analyses performed in which we include these covariates did yield similar estimates (Supplementary data Tables S4 and S5). In addition, other health-related variables included in the fully adjusted model such as alcohol consumption can be representative of health habits [36, 37]. Also, participation rate at baseline was moderate (56%), which may increase the risk of selection bias. Nevertheless, this issue is compensated through the use of refresher samples [13]. Similarly, there is still a chance for some attrition bias which migh hamper the accuracy of our estimations, but the average retention rate in SHARE (81%) and refresher participants considerably reduces such posibility [13]. Lastly, the use of a proxy for assessing the outcome variable might lead to a certain degree of loss of information and misclassification in such variable. However, as prior research has observed [38], a death proxy is a robust substitute to identify death in adult populations when fact of death is not available, which along with the high SHARE retention rate and the use of refresher samples make the chance of both selection and misclassification bias concerning death low. Our sensitivity analyses showed differences regarding handgrip strength between eligible participants with missing and nonmissing death cause, but these might be due to the age difference (i.e. missing participants were older, which possibly explain lower handgrip strength values) which, in turn, may also indicate that older participants are more likely to lose death cause information because of a reduced proxy network. Nonetheless, we included a weight variable in the analyses, which repairs both nonresponse and attrition.

## Conclusion

There is a linear inverse association between incremental levels of handgrip strength and risk of all-cause mortality in older adults up to 42 kg for men and 25 kg for women. Such linear association was observed for all-values within a specific handgrip strength range among individuals aged 65 and over for both sexes. By contrast, there is no clear association of handgrip strength with cancer mortality, but future investigation considering different cancer types is warranted.

## Supplementary Material

aa-21-2136-File003_afac117

## References

[1] Bohannon RW . Grip strength: an indispensable biomarker for older adults. Clin Interv Aging 2019; 14: 1681–91. 31631989 10.2147/CIA.S194543 PMC6778477

[2] Rantanen T, Masaki K, He Q, Ross GW, Willcox BJ, White L. Midlife muscle strength and human longevity up to age 100 years: a 44-year prospective study among a decedent cohort. Age (Omaha) 2012; 34: 563–70. 10.1007/s11357-011-9256-y PMC3337929 21541735

[3] Rantanen T, Masaki K, Foley D, Izmirlian G, White L, Guralnik JM. Grip strength changes over 27 yr in Japanese-American men. J Appl Physiol 1998; 85: 2047–53. 9843525 10.1152/jappl.1998.85.6.2047

[4] Kim GR, Sun J, Han M, Park S, Nam CM. Impact of handgrip strength on cardiovascular, cancer and all-cause mortality in the Korean longitudinal study of ageing. BMJ Open 2019; 9: e027019. 10.1136/bmjopen-2018-027019 PMC6527975 31072857

[5] Syddall H, Cooper C, Martin F, Briggs R, Sayer AA. Is grip strength a useful single marker of frailty? Age Ageing 2003; 32: 650–6. 14600007 10.1093/ageing/afg111

[6] García-Hermoso A, Cavero-Redondo I, Ramírez-Vélez R et al. Muscular strength as a predictor of all-cause mortality in an apparently healthy population: a systematic review and meta-analysis of data from approximately 2 million men and women. Arch Phys Med Rehabil 2018; 99: 2100–2113.e5. 29425700 10.1016/j.apmr.2018.01.008

[7] Celis-Morales CA, Welsh P, Lyall DM et al. Associations of grip strength with cardiovascular, respiratory, and cancer outcomes and all cause mortality: prospective cohort study of half a million UK Biobank participants. BMJ 2018; 361: k1651. 29739772 10.1136/bmj.k1651 PMC5939721

[8] Leong DP, Teo KK, Rangarajan S et al. Prognostic value of grip strength: findings from the Prospective Urban Rural Epidemiology (PURE) study. Lancet 2015; 386: 266–73. 25982160 10.1016/S0140-6736(14)62000-6

[9] Zhuang C, Zhang F, Li W et al. Associations of low handgrip strength with cancer mortality: a multicentre observational study. J Cachexia Sarcopenia Muscle 2020; 11: 1476–86. 32910535 10.1002/jcsm.12614 PMC7749566

[10] Yates T, Zaccardi F, Dhalwani NN et al. Association of walking pace and handgrip strength with all-cause, cardiovascular, and cancer mortality: a UK Biobank observational study. Eur Heart J 2017; 38: 3232–40. 29020281 10.1093/eurheartj/ehx449 PMC5837337

[11] Lee J . Associations between handgrip strength and disease-specific mortality including cancer, cardiovascular, and respiratory diseases in older adults: a meta-analysis. J Aging Phys Act 2020; 28: 320–31. 31810062 10.1123/japa.2018-0348

[12] Bergmann M, Scherpenzeel A, Börsch-Supan A. SHARE Wave 7 Methodology: Panel Innovations and Life Histories, Munich: Munich Center for the Economics of Aging (MEA), 2019.

[13] Börsch-Supan A, Brandt M, Hunkler C et al. Data resource profile: the Survey of Health, Ageing and Retirement in Europe (SHARE). Int J Epidemiol 2013; 42: 992–1001. 23778574 10.1093/ije/dyt088 PMC3780997

[14] Börsch-Supan A . Survey of Health, Ageing and Retirement in Europe (SHARE) Wave 7. Release version: 7.1.1. SHARE-ERIC 2020. http://www.share-project.org/data-documentation/waves-overview/wave-7.html.

[15] von Elm E, Altman DG, Egger M, Pocock SJ, Gøtzsche PC, Vandenbroucke JP. The strengthening the reporting of observational studies in epidemiology (STROBE) Statement: guidelines for reporting observational studies. Int J Surg 2014; 12: 1495–9. 25046131 10.1016/j.ijsu.2014.07.013

[16] Budziareck MB, Pureza Duarte RR, Barbosa-Silva MCG. Reference values and determinants for handgrip strength in healthy subjects. Clin Nutr 2008; 27: 357–62. 18455840 10.1016/j.clnu.2008.03.008

[17] Bhaskaran K, Leon DA, Douglas IJ, Smeeth L. Association of BMI with overall and cause-specific mortality : a population-based cohort study of 3 · 6 million adults in the UK. Lancet Diabetes Endocrinol 2018; 6: 944–53. 30389323 10.1016/S2213-8587(18)30288-2 PMC6249991

[18] Carioli G, Malvezzi M, Bertuccio P et al. European cancer mortality predictions for the year 2021 with focus on pancreatic and female lung cancer. Ann Oncol 2021; 32: 478–87. 33626377 10.1016/j.annonc.2021.01.006

[19] Rumgay H, Shield K, Charvat H et al. Articles Global burden of cancer in 2020 attributable to alcohol consumption : a population-based study. The Lancet Oncology 2020; 1071–80. 10.1016/S1470-2045(21)00279-5 PMC8324483 34270924

[20] Larney S, Tran LT, Hons B et al. All-cause and cause-specific mortality among people using extramedical opioids a systematic review and meta-analysis. JAMA Psychiatry 2020; 77: 493–502. 31876906 10.1001/jamapsychiatry.2019.4170 PMC6990804

[21] Harrell FE . Regression Modeling Strategies. New York, NY: Springer New York, 2001.

[22] Arvandi M, Strasser B, Meisinger C et al. Gender differences in the association between grip strength and mortality in older adults: results from the KORA-age study. BMC Geriatr 2016; 16: 1–8. 27903239 10.1186/s12877-016-0381-4 PMC5131446

[23] Taekema DG, Ling CHY, Blauw GJ et al. Circulating levels of IGF1 are associated with muscle strength in middle-aged- and oldest-old women. Eur J Endocrinol 2011; 164: 189–96. 21135066 10.1530/EJE-10-0703

[24] Bae E-J, Park N-J, Sohn H-S, Kim Y-H. Handgrip strength and all-cause mortality in middle-aged and older Koreans. Int J Environ Res Public Health 2019; 16: 740. 10.3390/ijerph16050740 PMC6427792 30823660

[25] Lera L, Albala C, Leyton B et al. Reference values of hand-grip dynamometry and the relationship between low strength and mortality in older Chileans. Clin Interv Aging 2018; 13: 317–24. 29503536 10.2147/CIA.S152946 PMC5826209

[26] Al SS, Markides KS, Ray L, Ostir GV, Goodwin JS. Handgrip strength and mortality in older Mexican Americans. J Am Geriatr Soc 2002; 50: 1250–6. 12133020 10.1046/j.1532-5415.2002.50312.x

[27] Koopman JJE, van Bodegom D, van Heemst D, Westendorp RGJ. Handgrip strength, ageing and mortality in rural Africa. Age Ageing 2015; 44: 465–70. 25331975 10.1093/ageing/afu165 PMC4411221

[28] Wong MD, Chung AK, Boscardin WJ et al. The contribution of specific causes of death to sex differences in mortality. Public Health Rep 2006; 121: 746–54. 17278410 10.1177/003335490612100615 PMC1781916

[29] Leong DP, Teo KK, Rangarajan S et al. Reference ranges of handgrip strength from 125,462 healthy adults in 21 countries: a prospective urban rural epidemiologic (PURE) study. J Cachexia Sarcopenia Muscle 2016; 7: 535–46. 27104109 10.1002/jcsm.12112 PMC4833755

[30] Tomasetti C, Li L, Vogelstein B. Stem cell divisions, somatic mutations, cancer etiology, and cancer prevention. Science (80- ) 2017; 355: 1330–4. 10.1126/science.aaf9011 PMC5852673 28336671

[31] Wiegert EVM, da Silva NF, de Oliveira LC, Calixto-Lima L. Reference values for handgrip strength and their association with survival in patients with incurable cancer. Eur J Clin Nutr 2021; 76: 93–102. 10.1038/s41430-021-00921-6 33911207

[32] Smith L, Yang L, Hamer M. Handgrip strength, inflammatory markers, and mortality. Scand J Med Sci Sports 2019; 29: 1190–6. 30972827 10.1111/sms.13433

[33] Il’yasova D, Colbert LH, Harris TB et al. Circulating levels of inflammatory markers and cancer risk in the health aging and body composition cohort. Cancer Epidemiol Biomarkers Prev 2005; 14: 2413–8. 16214925 10.1158/1055-9965.EPI-05-0316

[34] Watson J, Salisbury C, Banks J, Whiting P, Hamilton W. Predictive value of inflammatory markers for cancer diagnosis in primary care: a prospective cohort study using electronic health records. Br J Cancer 2019; 120: 1045–51. 31015558 10.1038/s41416-019-0458-x PMC6738065

[35] Alley DE, Crimmins E, Bandeen-Roche K, Guralnik J, Ferrucci L. Three-year change in inflammatory markers in elderly people and mortality: the Invecchiare in Chianti Study. J Am Geriatr Soc 2007; 55: 1801–7. 17727645 10.1111/j.1532-5415.2007.01390.x PMC2646097

[36] Beard E, West R, Michie S, Brown J. Association between smoking and alcohol-related behaviours: a time–series analysis of population trends in England. Addiction 2017; 112: 1832–41. 28556467 10.1111/add.13887 PMC5600127

[37] Valencia-Martín JL, Galán I, Rodríguez-Artalejo F. The association between alcohol consumption patterns and adherence to food consumption guidelines. Alcohol Clin Exp Res 2011; 35: 2075–81. 21848958 10.1111/j.1530-0277.2011.01559.x

[38] Mealing NM, Dobbins TA, Pearson SA. Validation and application of a death proxy in adult cancer patients. Pharmacoepidemiol Drug Saf 2012; 21: 742–8. 22020956 10.1002/pds.2257

