# Supplementary material for: Associations of handgrip strength with all-cause and cancer mortality in older adults: a prospective cohort study in 28 countries
Source: Age Ageing. 2022 May 25;51(5):afac117. doi: 10.1093/ageing/afac117 (PMC9351371; doi:10.1093/ageing/afac117)
Supplement: aa-21-2136-File003_afac117 [file aa-21-2136-file003_afac117.zip › aa-21-2136-File003_afac117.docx]

**Associations of handgrip strength with all-cause and cancer mortality in older adults: A** **prospective cohort study in 28 countries**

Supplementary Data

**Table 1.** Country weights for study participants stratified by sex

**Figure 1.** Simplified DAG used for the selection of covariables for the association between handgrip and cancer mortality

**Table 2.** E-Values for the association of handgrip strength with all-cause mortality

**Table 3.** E-Values for significant associations of handgrip strength with cancer mortality

**Table 4.** Association of handgrip strength with all-cause mortality after removing participants who died within the first two years of follow-up

**Table 5.** Association of handgrip strength with cancer mortality after removing participants who died because of cancer within the first two years of follow-up

**Table 6.** Mean values (SD) of relevant continuous variables for eligible participants with missing and non-missing information on death cause at study entry

**Figure 2**. Evolution of the sensibility and specificity in relation to different handgrip strength cut-off points in men

**Figure 3.** Evolution of the sensibility and specificity in relation to different handgrip strength cut-off points in women

**Figure 4.** Dose–response association between handgrip strength and all-cause mortality in middle-aged men

**Figure 5.** Dose–response association between handgrip strength and all-cause mortality in middle-aged women

**Figure 6.** Dose–response association between handgrip strength and all-cause mortality in older men

**Figure 7.** Dose–response association between handgrip strength and all-cause mortality in older women

**Table 7**. Prospective associations between handgrip strength and handgrip strength relative to body mass index with all cause and cancer mortality

**Figure 8.** Dose–response association (Adjusted hazard ratios and associated 95% confidence interval band) between handgrip strength (kg) and all-cause mortality in men with underweight and normal BMI (body mass index)

**Figure 9**. Dose–response association (Adjusted hazard ratios and associated 95% confidence interval band) between handgrip strength (kg) and all-cause mortality in women with underweight and normal BMI (body mass index)

**Figure 10**. Dose–response association (Adjusted hazard ratios and associated 95% confidence interval band) between handgrip strength (kg) and all-cause mortality in men with overweight and obesity according to BMI (body mass index)

**Figure 11.** Dose–response association (Adjusted hazard ratios and associated 95% confidence interval band) between handgrip strength (kg) and all-cause mortality in women with overweight and obesity according to BMI (body mass index)

**Figure 12**. Overall trajectories for median values of handgrip strength in relation to outcome (all-cause mortality)

**Figure 13.** Overall trajectories for median values of handgrip strength in relation to outcome (cancer mortality)

**Appendix 1.** Details on methods and study design

**Appendix 2.** Details on study variables

**Appendix 3.** Sensitivity analyses

**Appendix 4.** Results of sensitivity analyses

| **Table 1. Country weights for study participants stratified by sex^a^** | | |
| --- | --- | --- |
|  | **Men** | **Women** |
| **Country** |  |  |
| 11 Austria | 3,971,306 | 4,200,660 |
| 23 Belgium | 5,140,743 | 5,337,874 |
| 51 Bulgaria | 3,727,030 | 3,931,942 |
| 47 Croatia | 2,035,607 | 2,234,538 |
| 28 Czech Republic | 18,478,468 | 19,686,977 |
| 18 Denmark | 2,684,213 | 2,735,229 |
| 35 Estonia | 629,579 | 725,196 |
| 55 Finland | 2,567,723 | 2,678,763 |
| 17 France | 30,587,080 | 32,592,276 |
| 12 Germany | 40,296,958 | 4,2172,474 |
| 19 Greece | 5,438,858 | 5,548,456 |
| 32 Hungary | 4,797,763 | 5,309,383 |
| 30 Ireland | 2,031,416 | 2,038,846 |
| 25 Israel | 3,418,244 | 3,511,856 |
| 16 Italy | 28,144,391 | 29,825,093 |
| 57 Latvia | 1,038,040 | 1,212,351 |
| 48 Lithuania | 1,543,478 | 1,759,050 |
| 31 Luxembourg | 4,786,760 | 5,300,395 |
| 59 Malta | 200,281 | 203,553 |
| 14 Netherlands | 8,092,628 | 8,227,240 |
| 29 Poland | 2,076,648 | 2,083,266 |
| 33 Portugal | 5,077,470 | 5,425,560 |
| 61 Romania | 10,391,006 | 10,928,679 |
| 63 Slovakia | 2,604,797 | 2,768,010 |
| 34 Slovenia | 978,181 | 1,022,293 |
| 15 Spain | 21,535,302 | 22,117,853 |
| 20 Switzerland | 3,640,621 | 3,796,494 |
| 13 Sweden | 4,477,600 | 4,551,972 |

^a^Year 2004

**Figure 1. Simplified DAG used for the selection of covariables for the association between handgrip and cancer mortality**

**
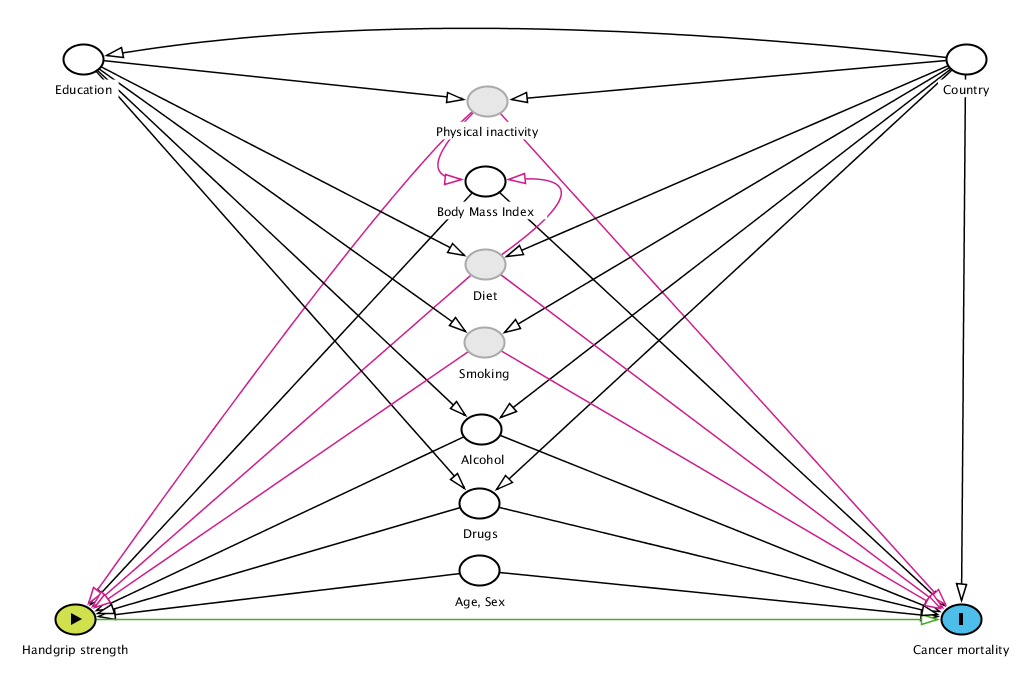
**

Nodes: Green (with ►) = exposure, Blue (with I) = outcome, Red = ancestor of exposure and outcome (confounder), White = adjusted variable, Grey = unobserved (latent) variable.

Arrows: Green = causal path, red = biasing path, black = blocked path

| **Table 2. E-Values for the association of handgrip strength with all-cause mortality** | | |
| --- | --- | --- |
| **Subgroup** | **Handgrip strength*** | **E-Value** |
|  |  | **(95% CI Lower Limit)** |
| Men | First third | reference |
|  | Second third | 2.97 (2.30) |
|  | Third third | 3.60 (2.78) |
| Women | First third | reference |
|  | Second third | 2.72 (1.96) |
|  | Third third | 3.93 (2.66) |
| *Model B (Fully adjusted model; adjusted for age, education, country, body mass index, drug and alcohol consumption) + exclusions of all-cause deaths during the first two years of follow-up  The E-value is the minimum strength of association on the hazard ratio scale that an unmeasured confounder would need to have with both the treatment and the outcome to explain away an exposure-outcome association.   \| **Table 3. E-Values for significant associations of handgrip strength with cancer mortality** \| \| \| \| --- \| --- \| --- \| \| **Subgroup** \| **Handgrip strength*** \| **E-Value** \| \| **(95% CI Lower Limit)** \| \| Women \| First third \| reference \| \|  \| Third third \| 2.72 (1.25) \| \| *Model B (Fully adjusted model; adjusted for age, education, country, body mass index, drug and alcohol consumption) + exclusions of cancer deaths during the first two years of follow-up  The E-value is the minimum strength of association on the hazard ratio scale that an unmeasured confounder would need to have with both the treatment and the outcome to explain away an exposure-outcome association. \| \| \| | | |

| **Table 4. Association of handgrip strength with all-cause mortality after removing participants who died within the first two years of follow-up** | | |
| --- | --- | --- |
| **Subgroup** | **Handgrip strength*** | **HR (95% CI)** |
| Men | First third | reference |
|  | Second third | 0.59 (0.40-0.89) |
|  | Third third | 0.19 (0.11-0.35) |
| Women | First third | reference |
|  | Second third | 0.53 (0.30-0.92) |
|  | Third third | 0.27 (0.12-0.59) |
| HR: Hazard Ratio; CI: Confidence Interval  *Fully additional adjusted model using Cox regression; adjusted for age, education, country, body mass index, alcohol consumption, smoking habits, fruits and vegTables consumption, and physical inactivity) + exclusions of all-cause deaths during the first two years of follow-up and de | | |

| **Table 5. Association of handgrip strength with cancer mortality after removing participants who died because of cancer within the first two years of follow-up** | | |
| --- | --- | --- |
| **Subgroup** | **Handgrip strength*** | **HR (95% CI)** |
| Men | First third | reference |
|  | Second third | 1.02(0.50-2.08) |
|  | Third third | 0.17(0.07-0.41) |
| Women | First third | reference |
|  | Second third | 0.23 (0.08-0.66) |
|  | Third third | 0.45 (0.17-1.21) |
| SHR: Hazard Ratio; CI: Confidence Interval  *Fully additional adjusted model using Cox regression; adjusted for age, education, country, body mass index, alcohol consumption, smoking habits, fruits and vegTables consumption, and physical inactivity) + exclusions of cancer deaths during the first two years of follow-up | | |

| **Table 6. Mean values (SD) of relevant continuous variables for eligible participants with missing and non-missing information on death cause at study entry** | | | | | | | | |
| --- | --- | --- | --- | --- | --- | --- | --- | --- |
|  | **Men** | | | | **Women** | | | |
|  | **Missing (n=63)** | **Non-missing (n=54 807)** | **Difference** | ***p**** | **Missing**  **(n=53)** | **Non-missing (n=66 576)** | **Difference** | ***p**** |
| **Age (y)** | **73.6(11.5)** | **64.0(9.6)** | **9.6** | **>0.01** | **78.6(9.1)** | **63.9(10.2)** | **14.7** | **>0.01** |
| **Body Mass Index (kg/m^2^)** | 26,9(3.8) | 27.2(4.1) | -0.3 | 0.56 | 27,4(4.2) | 26.7(5.0) | 0.7 | 0,31 |
| **Handgrip strength (kg)** | 36.2(10.4) | 42.6(11.1) | -6.4 | **>0.01** | 19.8(6.5) | 27.1(7.8) | -7.3 | **>0.01** |
| *p-significance estimated through a non-paired t-test | | | | | | | | |

**Figure 2.** **Evolution of the sensibility and specificity in relation to different handgrip strength cut-off points for all-cause mortality in men***


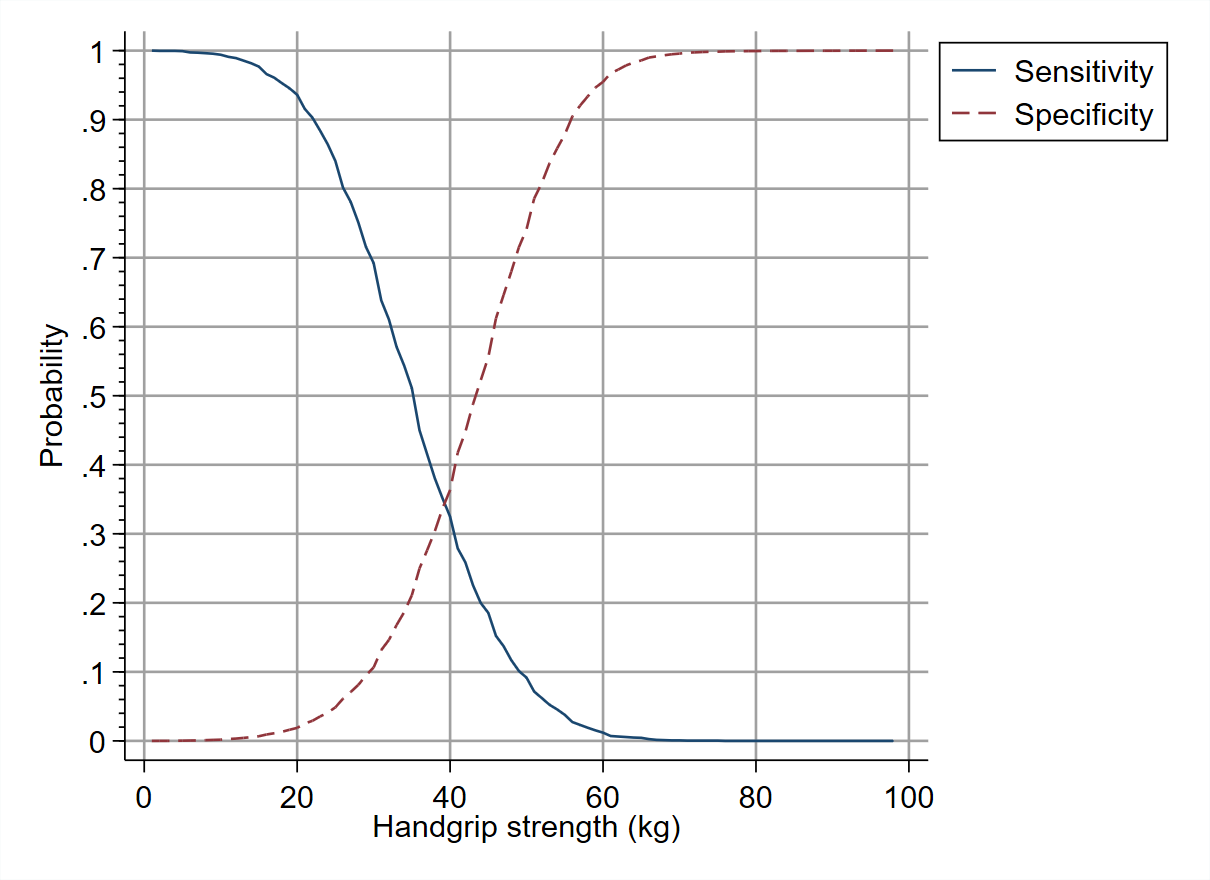


*Crude analyses

**Figure 3.** **Evolution of the sensibility and specificity in relation to different handgrip strength cut-off points for all-cause mortality in women***


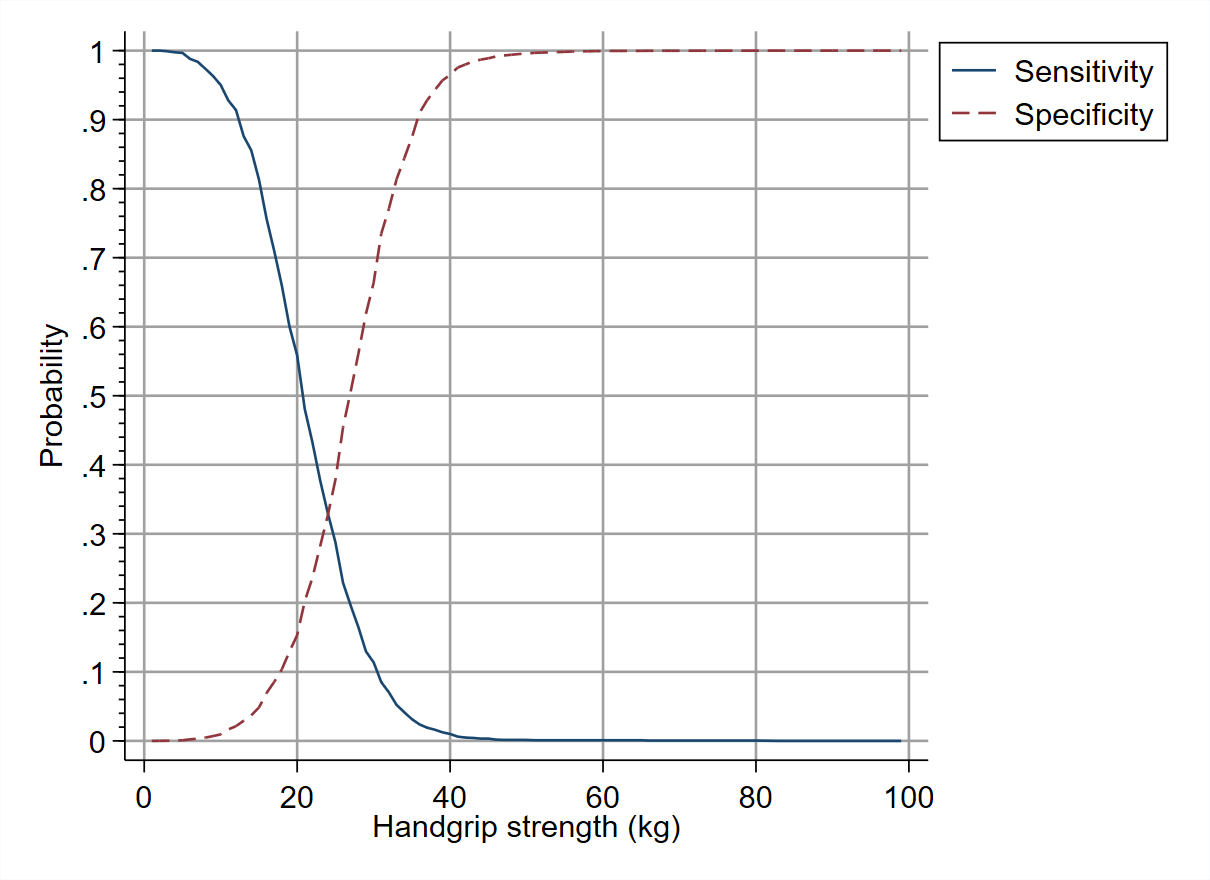


*Crude analyses

**Figure 4.** **Dose–response association (Adjusted hazard ratios and associated 95% confidence interval band) between handgrip strength (kg) and all-cause mortality in middle-aged men (<65 years). Adjusted for Model B (age, education, country, body mass index, drug and alcohol consumption) and exclusion of all-cause deaths of two first years of follow-up**


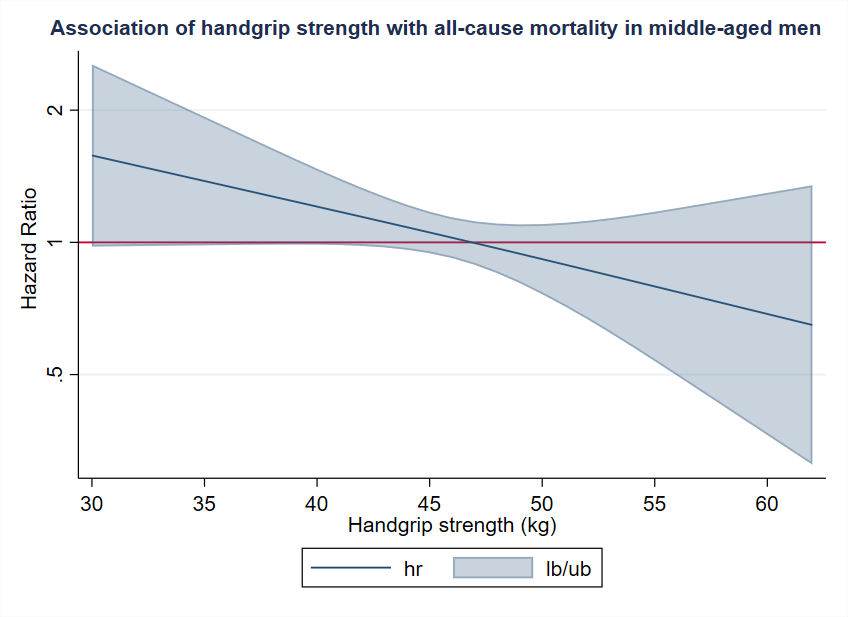


**Figure 5.** **Dose–response association (Adjusted hazard ratios and associated 95% confidence interval band) between handgrip strength (kg) and all-cause mortality in middle-aged women (<65 years). Adjusted for Model B (age, education, country, body mass index, drug and alcohol consumption) and exclusion of all-cause deaths of two first years of follow-up**


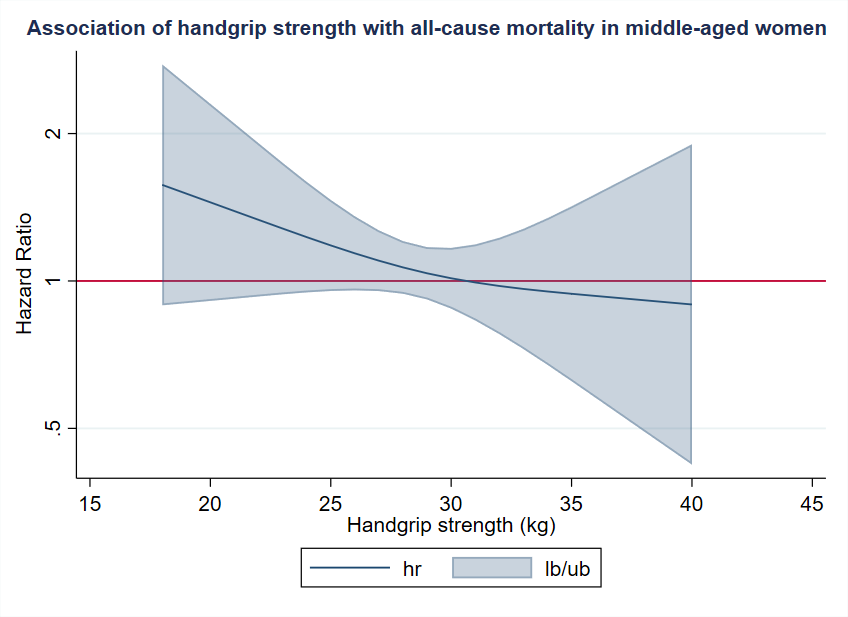


**Figure 6.** **Dose–response association (Adjusted hazard ratios and associated 95% confidence interval band) between handgrip strength (kg) and all-cause mortality in older men (≥65 years). Adjusted for Model B (age, education, country, body mass index, drug and alcohol consumption) and exclusion of all-cause deaths of two first years of follow-up**


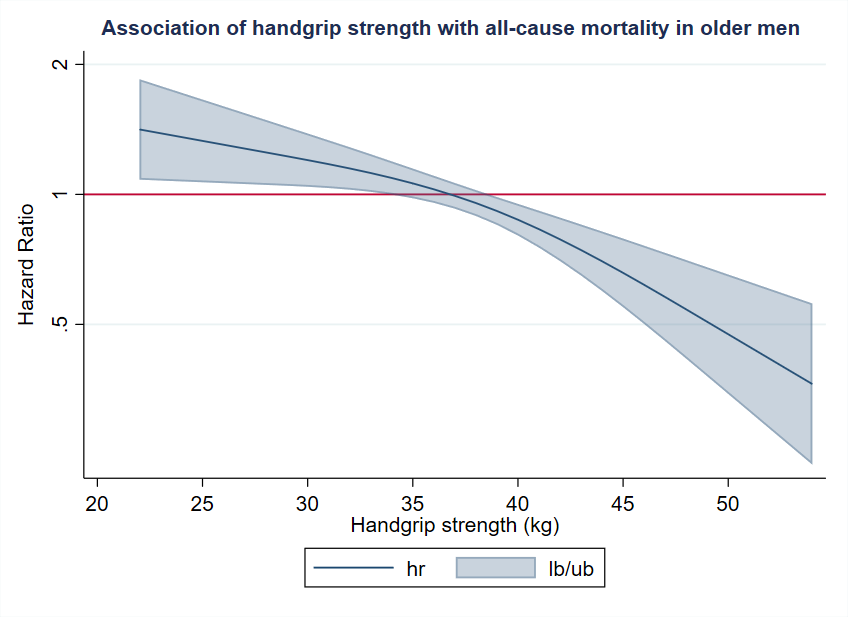


**Figure 7.** **Dose–response association (Adjusted hazard ratios and associated 95% confidence interval band) between handgrip strength (kg) and all-cause mortality in older women (≥65 years). Adjusted for Model B (age, education, country, body mass index, drug and alcohol consumption) and exclusion of all-cause deaths of two first years of follow-up**


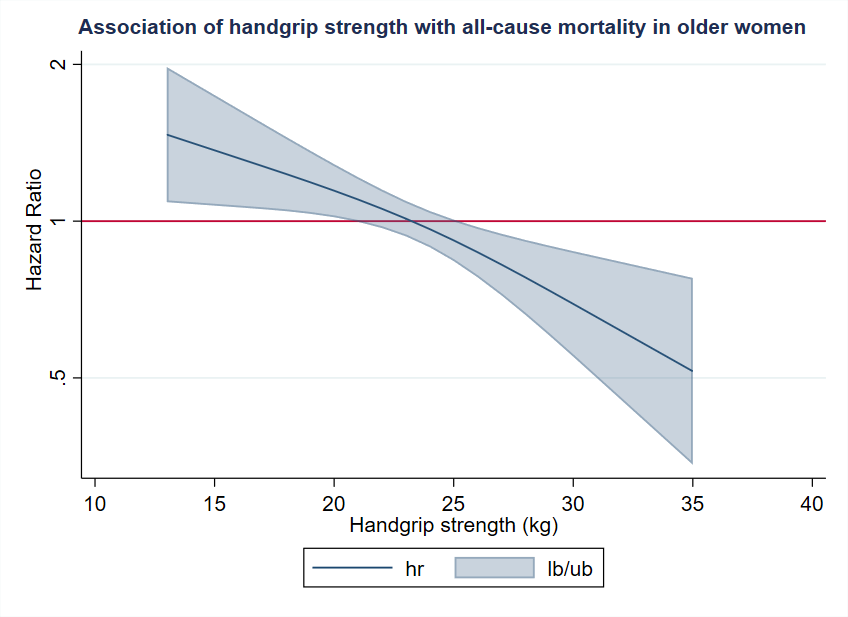


| **Table 7.** **Prospective associations between sex-standardized handgrip strength and sex-standardized handgrip strength relative to body mass index with all cause and cancer mortality (HR [95% CI])** | | |
| --- | --- | --- |
|  | **Sex-standardized handgrip strength** | **Relative sex-standardized handgrip strength*** |
| **All-cause mortality** | 0.98 (0.97-0.98) | 0.80 (0.76-0.85) |
| **Cancer mortality** | 1.00 (0.99-1.02) | 1.03 (0.93-1.14) |
| HR: Hazard Ratio; CI: Confidence Interval  *Handgrip strength divided by body mass index and thereafter standardized using sex-specific mean and standard deviation of the whole sample ([X − Mean] ÷ SD). Marginal structured model adjusted by age, country, and baseline, current and lagged education, drug and alcohol consumption | | |

**Figure 8. Dose–response association (Adjusted hazard ratios and associated 95% confidence interval band) between handgrip strength (kg) and all-cause mortality in men with underweight and normal BMI (body mass index). Adjusted for Model B (age, education, country, drug and alcohol consumption) and exclusion of all-cause deaths of two first years of follow-up**


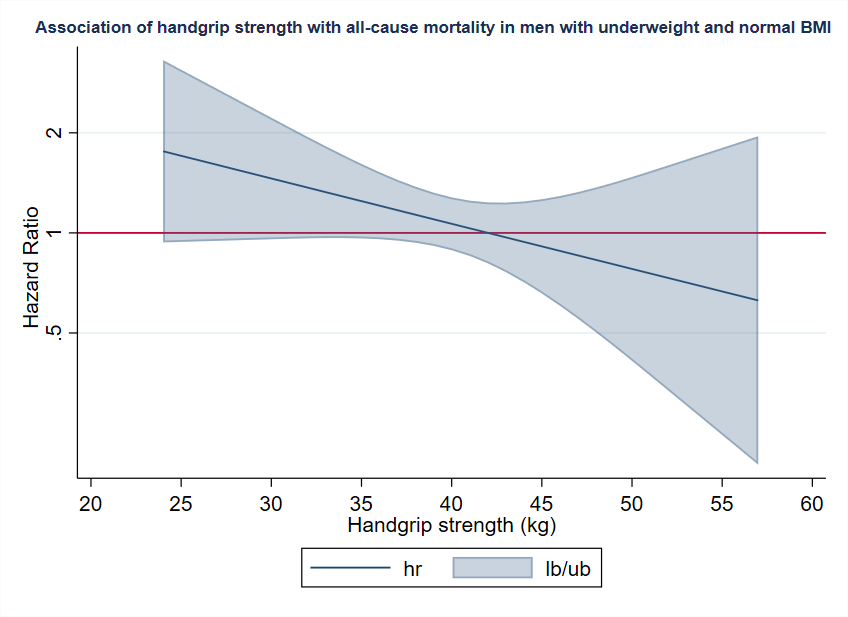


**Figure 9.** **Dose–response association (Adjusted hazard ratios and associated 95% confidence interval band) between handgrip strength (kg) and all-cause mortality in women with underweight and normal BMI (body mass index). Adjusted for Model B (age, education, country, drug and alcohol consumption) and exclusion of all-cause deaths of two first years of follow-up**

**
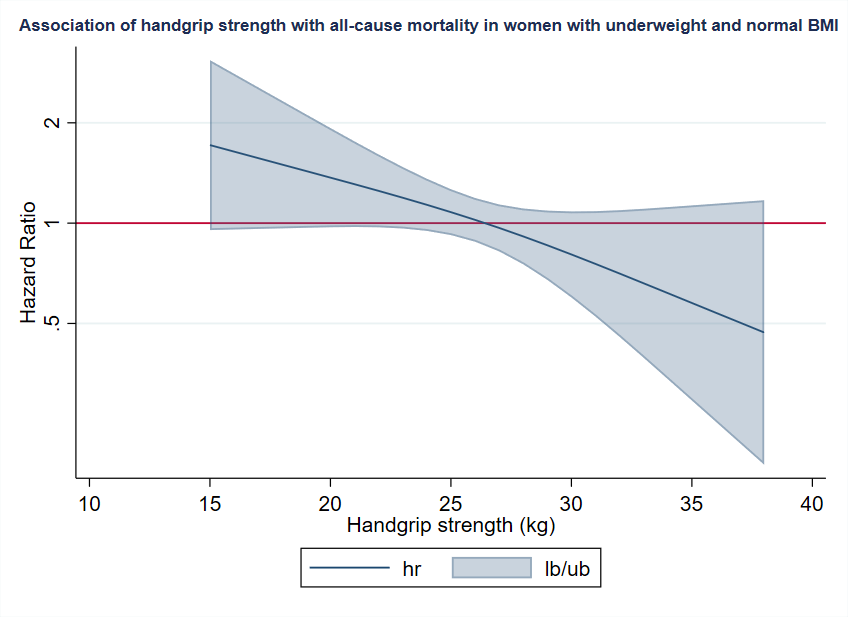
**

**Figure 10. Dose–response association (Adjusted hazard ratios and associated 95% confidence interval band) between handgrip strength (kg) and all-cause mortality in men with overweight and obesity according to BMI (body mass index). Adjusted for Model B (age, education, country, drug and alcohol consumption) and exclusion of all-cause deaths of two first years of follow-up**

**
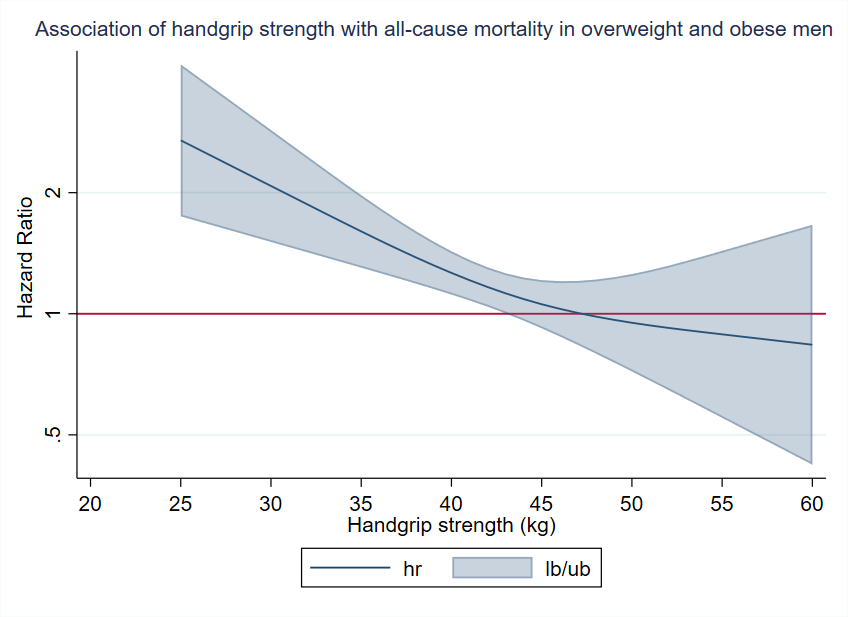
**

**Figure 11. Dose–response association (Adjusted hazard ratios and associated 95% confidence interval band) between handgrip strength (kg) and all-cause mortality in women with overweight and obesity according to BMI (body mass index). Adjusted for Model B (age, education, country, drug and alcohol consumption) and exclusion of all-cause deaths of two first years of follow-up**

**
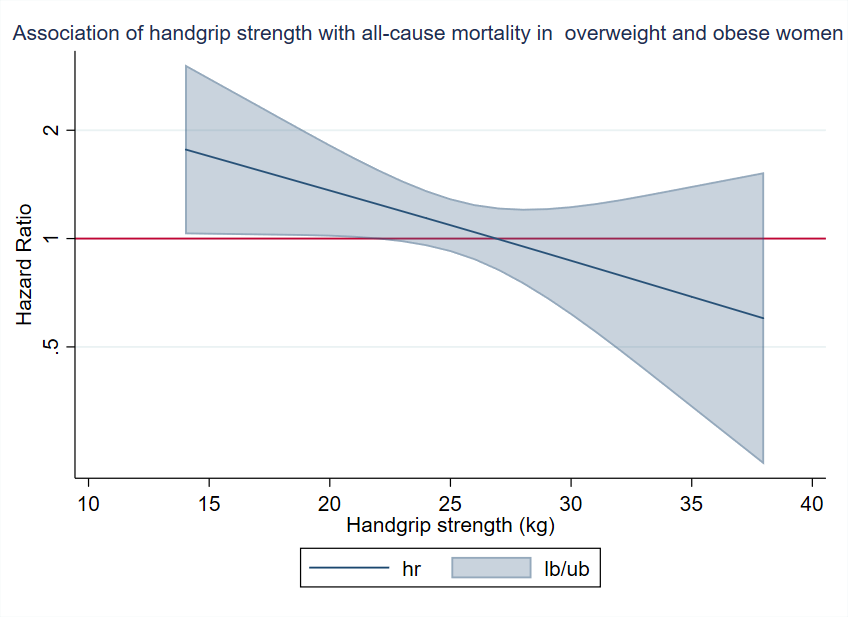
**

**Figure 12. Overall trajectories for median values of handgrip strength in relation to outcome (all-cause mortality)***


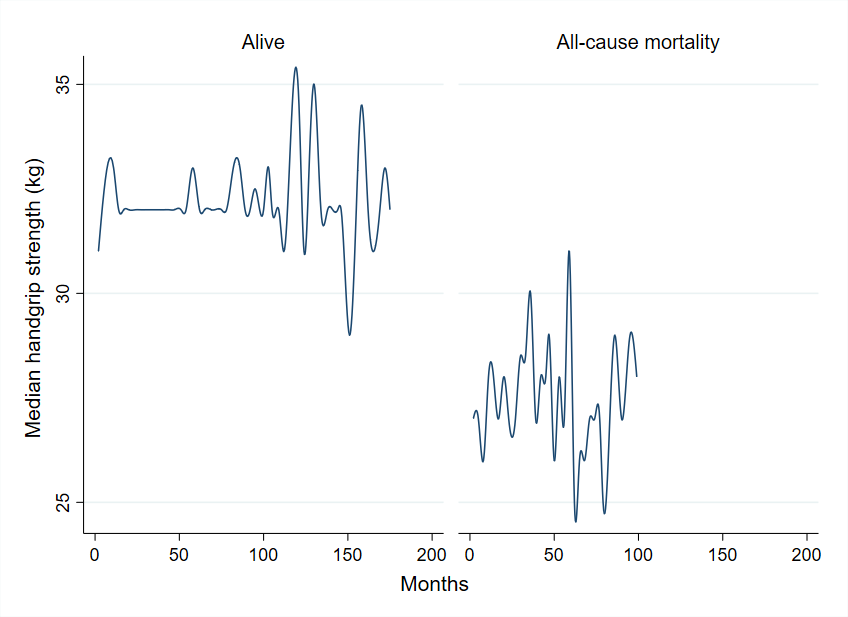


*Crude analyses

**Figure 13. Overall trajectories for median values of handgrip strength in relation to outcome (cancer mortality)***


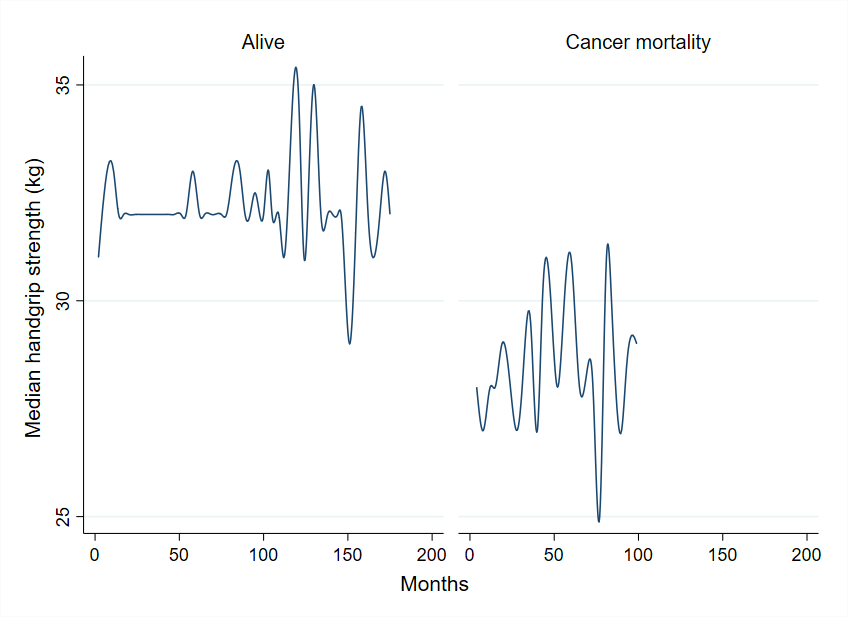


*Crude analyses

Appendix 1

Details of the study methods

Representativeness of SHARE waves is assured using a multi-stage stratified sampling design in which countries are divided into different strata according to their geographical area. Municipalities or zip codes within these strata served as primary sampling units. Data used in our study were collected through home computer-assisted personal interviews from February 2004 to January 2019. Data from SHARE were collected using ex-ante harmonized interviews; and new respondents were added in each wave to compensate for the attrition bias due to losses [1]. Of the original regular panel sample, only participants aged 50 years or older and that were free from any current or prior cancer diagnosis were included in the current study (n=131 861). Participants with no available data for any of the study variables were excluded from the analyses (n=10478).

Reference list

1. Börsch-Supan A. Survey of Health, Ageing and Retirement in Europe (SHARE) Wave 7. Release version: 7.1.1. SHARE-ERIC. 2020.

Appendix 2

Details of the study variables

According to the SHARE protocol, participants were instructed to set their elbow in a 90° angle flexion while either standing or sitting, neutral wrist position, and upper arm set in a vertical position against the trunk. Trained interviewers verbally encouraged participants with standardized instructions to grip with maximum effort for a few seconds. Handgrip strength was defined as the maximum value of either hand.

For specific cause of mortality, the following question was used: “What was the main cause of respondent’s death?” Possible answers comprised cancer, heart attack, stroke, other cardiovascular disease related illnesses (heart failure and arrhythmia), respiratory, digestive, or severe infectious disease, other causes, don’t know, or refusal. Less than 1% of the eligible participants had missing values on death cause, which was due to either lack of information or refusal of the proxy to disclose such information. For all-cause mortality, participants were categorized into 0 (alive) and 1(dead), whereas for specific cancer mortality participants were categorized into alive, death due to cancer, or death due to other causes.

Self-reported age and sex, country of residence at the time of interview, education, body mass index, and alcohol and drug consumption were identified as potential confounders. Education was self-reported by participants and thereafter coded using the 1997 version of the International Standard Classification of Education [2]. Body mass index was calculated from self-reported height and weight and subsequently grouped into 4 categories according to standards proposed by World Health Organization (WHO) [3]. Alcohol consumption was estimated through the following question: “How many days a week did you consume alcohol during the last six months?” and answers included the following possible options: “Almost every day”, Five or six days a week, “Three or four days a week”, “Once or twice a week”, “Once or twice a month”, “Less than once a month”, “Not at all in the last 6 months”, “Refusal to answer”, or “Don´t know”. Drug consumption (i.e., medicines for treating chronic conditions) was assessed through the following question: “Do you currently use drugs at least once a week for problems mentioned on this card?” This variable was re-coded into the categories “None” for those who answered such option in the survey, and “Any” for those who took one or more of a list drugs [1].

Reference list

1. Börsch-Supan A. Survey of Health, Ageing and Retirement in Europe (SHARE) Wave 7. Release version: 7.1.1. SHARE-ERIC. 2020.

2. UNESCO. International Standard Classification of Education, ISCED 1997. In: Advances in Cross-National Comparison. Boston, MA: Springer US, 2003: 195–220.

3. World Health Organization (WHO). Obesity : preventing and managing the global epidemic. Geneva, 2000.

Appendix 3

Sensitivity analyses

To minimize the potential influence of reverse causality, we conducted sensitivity analyses excluding participants who died due to all-causes and due to cancer within the first 2 years of follow-up for all-cause and cancer specific mortality respectively. Unmeasured confounding was assessed through E-Values in the fully adjusted model with participants who died within the two first years excluded. An additional model including other potential confounders such as smoking habits, diet, and physical inactivity was also examined. To assess differences between participants with missing and non-missing values on cause of death we checked mean values of relevant variables such as age, body mass index, and handgrip strength for the two cases. Crude optimal cut-off points for ROC curves and sensitivity and specificity curves were inspected to further examining of thresholds of handgrip strength in our study. Moreover, in order to control for time-varying confounding as well as to examine relative handgrip strength, we conducted a marginal structured model using sex-standardized and relative sex-standardized handgrip strength (i.e., handgrip strength divided by body mass index and thereafter standardized using sex-specific mean and standard deviation of the whole sample ([X − Mean] ÷ SD)) as exposures. Furthermore, dose-response analyses using restricted cubic splines stratified by body mass index (i.e., underweight/normal weight vs overweight/obese) were carried out. In addition, median handgrip strength trajectories based on the outcome were also examined. Finally, we examined continuous handgrip strength additionally stratified by mean age using restricted cubic splines.

Appendix 4

Results of sensitivity analyses

Results of sensitivity analyses excluding all-cause and cancer deaths occurring within the first 2 years of follow-up differed substantially from those of the main analysis (Table 4, Table 5). Men and women in the middle of handgrip strength showed attenuated but still significantly lower risk of all-cause mortality compared with the first third (i.e., reference). On the other hand, the association between handgrip strength and cancer mortality was attenuated to such an extent that significant associations were only detected for women from the highest third. Moreover, slight variations concerning optimal thresholds for all-cause mortality were observed for both sexes (Figure 2, Figure 3); additional age-stratified analyses showed similar but non-significant associations for middle-aged men and women (<65 years), whereas significant linear dose-response associations were identified for older men and women (≥65 years) (Figure 4, Figure 5, Figure 6, Figure 7). We detected differences between eligible participants with missing and non-missing values on the cause of death for age and handgrip strength (Table 6). Controlling for time-varying confounding using marginal structured modelling confirmed both the significant inverse association between handgrip strength and all-cause mortality and the no significant association between handgrip strength and cancer mortality (Table 7). Dose-response analyses stratified by two categories of body mass index within sexes confirmed the observed thresholds for the category of overweight and obese individuals solely (Figure 8, Figure 9, Figure 10, Figure 11). Handgrip strength trajectories showed lower median values for either all-cause or cancer mortality outcomes than for their counterparts, although substantial fluctuations over these values were identified, particularly at the end of the follow-up period (Figure 12, Figure 13).
